# Attitudes of cardiac rehabilitation and stroke teams towards integration of stroke survivors into adapted cardiac rehabilitation: A focus group study

**DOI:** 10.1177/02692155241253476

**Published:** 2024-05-15

**Authors:** Nicola Clague-Baker, Thompson Robinson, Annegret Hagenberg, Sophie Drewry, Sally Singh

**Affiliations:** 1 Physiotherapy Department, School of Allied Health Professions and Nursing, 4591 University of Liverpool, Liverpool, UK; 2 Department of Cardiovascular Sciences and NIHR Leicester Biomedical Research Centre, 4488 University of Leicester, Leicester, UK; 3 Department of Health and Social Affairs, 52775 Hochschule Fresenius University of Applied Sciences, Munich, Germany; 4 Physiotherapy Department, 4488 University of Leicester, Leicester, UK; 5 Department of Respiratory Science, 4488 University of Leicester, Leicester, UK

**Keywords:** Cardiac rehabilitation, stroke, beliefs, lifestyle modification

## Abstract

**Objective:**

To explore the attitudes and beliefs of cardiac rehabilitation and stroke teams towards adapted cardiac rehabilitation, and the broader topics of exercise, healthy lifestyles and health behaviour change, for people with mild-to-moderate severity stroke in the sub-acute phase of recovery.

**Design:**

Qualitative focus group-based study.

**Setting:**

Acute and community national health service trusts.

**Participants:**

Stroke and cardiac rehabilitation team members.

**Intervention:**

Adapted cardiac rehabilitation.

**Main measures:**

Focus groups. Thematic analysis was applied to the transcribed data.

**Results:**

Overall, 57 health professionals participated in 12 focus groups. Positive impacts for teams and stroke survivors were identified particularly confidence. However, there were negatives, barriers and adaptations identified. In addition, there was a lack of knowledge for cardiac rehabilitation teams in relation to stroke survivors and stroke teams in relation to cardiac rehabilitation, exercise and healthy lifestyles.

**Conclusions:**

Cardiac rehabilitation and stroke staff attitudes to cardiac rehabilitation for stroke survivors showed a range of benefits, negatives, barriers and adaptations needed. Confidence and knowledge of the cardiac rehabilitation and stroke teams needs to be addressed.

**Registration:**

ISRCTN65957980.

## Introduction

There are over 12.2 million new strokes worldwide each year,^1^ up to one third being recurrent.^2^ The Royal College of Physicians^3^ state that lifestyle modifications ‘could deliver a greater than 80% risk reduction’ when combined with secondary prevention measures. However, a number of studies have found that stroke survivors are not aware of their lifestyle risks,^4,5^ do not know how to implement lifestyle change, nor identify many barriers to such change.^6–9^

The UK Cardiovascular Disease Outcomes Strategy^10^ and more recently the National Clinical Guideline for Stroke^3^ recommend the use of existing cardiac rehabilitation programmes post-transient ischaemic attack and for mildly disabled stroke survivors in order to address secondary prevention and potentially reduce cardiovascular risk. However, this assumes that existing cardiac rehabilitation teams have the knowledge and expertise to support stroke survivors. A search of the literature found that there were no identified post-stroke studies exploring staff attitudes towards stroke survivors attending cardiac rehabilitation. Research that was found related to stroke staff attitudes towards *exercise* post-stroke showed that those staff with positive beliefs of the benefits of exercise and positive exercise behaviours were more likely to recommend exercise to patients.^11^ A systematic review of 20 studies concluded that training was needed for stroke staff related to aerobic exercise in order to increase confidence and knowledge,^12^ this message was mirrored in a survey-based study in Canada.^13^ As cardiac rehabilitation additionally provides healthy lifestyle advice a literature search was also carried out related to staff attitudes to healthy lifestyle advice post-stroke. It was found that there was limited research exploring stroke staff attitudes to lifestyle modification including exercise post-stroke, and limited staff knowledge of healthy behaviours, with exercise rarely encouraged.^14^ However, one recent interview-based study with eight stroke survivors^15^ identified the importance of staff being aware of individualised health coaching post-stroke focussing on emotional as well as physical health.

In summary, there is no research exploring healthcare professionals’ attitudes toward adapted cardiac rehabilitation for stroke survivors and limited evidence exploring their attitudes to lifestyle modification post-stroke. The authors of this study, therefore, aimed to explore the attitudes and beliefs of cardiac rehabilitation and stroke teams towards adapted cardiac rehabilitation. In addition, the aim was to explore the broader topics of exercise, healthy lifestyle and health behaviour change for people with mild to moderate severity, up to six months post-stroke or transient ischaemic attack.

## Method

### Ethical approval

The study was approved by the National Research Ethics Service Committee East Midlands (Reference 14/EM/1067).

### Participants

Using a phenomenological, qualitative interpretive approach with five researchers, seven focus groups with cardiac rehabilitation and stroke teams were conducted prior to stroke survivors taking part in adapted cardiac rehabilitation and five focus groups after taking part in adapted cardiac rehabilitation. Managers acted as the gatekeepers so participants were not approached directly. Focus groups were conducted across three hospital sites, and participants were recruited using purposive and convenience sampling. Included were members of cardiac rehabilitation and stroke teams, as a therapist or therapy assistant, nurse or doctor. A range of experience and age was sought within a large UK University Teaching Hospital National Health Service Trust.

All of the cardiac rehabilitation team members involved in the focus groups had been actively involved in the adapted cardiac rehabilitation. None of the stroke team members involved in the focus groups were involved in the adapted cardiac rehabilitation. The three facilitators of the focus groups were the specialist stroke physiotherapists involved in the adapted cardiac rehabilitation.

The previous adapted cardiac rehabilitation study has been published separately,^16^ the study introduced 32 stroke survivors into adapted cardiac rehabilitation twice a week for 6 weeks. The participants had a mean age of 64.4 years and a median stroke severity of 2 using the National Institutes of Health Stroke Scale^17^ (range: 0–6) and all were within 6 months of having had a stroke.

### Procedure

Open trigger questions were developed by the research group (see Table 1). A pilot focus group confirmed the choices. Focus groups were arranged at the convenience of the staff in hospital meeting rooms. For practical reasons related to location, speciality focus groups (cardiac rehabilitation and stroke) were conducted separately. Other than the researchers and participants there were no other people in the focus groups.

**Table 1.** Sample questions for focus groups with cardiac rehabilitation and stroke staff.

Question:
Based on your experience how do patients participate in exercise before and after a stroke or TIA?
What do you see as the positives of patients exercising after a stroke or TIA?
Do you think there are any negatives of patients exercising after a stroke or TIA?
What do you see as the barriers to stroke or TIA patients exercising after a stroke or TIA?
Inclusion and exclusion criteria for trial – any thoughts?
Do you think the cardiac rehabilitation programmes had a positive or negative effect on the patients who have had a stroke or TIA?
Do you have any concerns about people with stroke attending cardiac rehabilitation?
How would you describe your role and responsibility in influencing patients to take up a healthy lifestyle after a stroke or TIA?
Do you think you have enough knowledge to provide the healthy lifestyle messages?
In your experience what are patients’ attitudes to health behaviour change after a TIA or stroke?
Do you have any other thoughts about stroke and TIA patients and exercise, cardiac rehabilitation or healthy lifestyles?

TIA – Transient Ischaemic Attack.

Focus groups were facilitated by the principal investigator, female physiotherapy lecturer/practitioner (NCB, MPhil), with the assistance of two female research physiotherapists (AH, MSc and SD, MSc). All three researchers had completed qualitative masters level research and were supported by a specialist qualitative researcher from Coventry University (CC, PhD). NCB, AH and SD all took part in the adapted cardiac rehabilitation groups as specialist stroke physiotherapists and were known to the stroke and cardiac rehabilitation teams prior to the start of the study. The participants were aware of the reasons for the research. As all three researchers were stroke physiotherapists, they reflected on this before and after each focus group to ensure they reduced their influence on the data collection.

Discussions were summarised at the end of each focus group to allow the participants to add any final observations. Field notes were written during and after each focus group. There were no follow-up interviews. All focus groups were audiotaped and transcribed verbatim by a professional, independent transcriber. Data collection, and the initial stages of data analysis, occurred concurrently, and the schedule of questions were expanded after each focus group as more topics were explored.

### Data analysis

Thematic analysis was conducted according to the analytic framework developed by Braun and Clarke^18^ involving five qualitative researchers (NCB, AH, SD and two specialist qualitative researchers from the universities of Coventry and Leicester, CC and SW). Stroke team and cardiac rehabilitation team focus group data were analysed together. Codes were identified by each researcher individually according to common subjects identified, compared across the team and expanded accordingly. No software was used for this process. Themes were identified that captured the essence of these codes. The principal investigator used a reflexive diary and bracketing,^18^ ensuring an awareness of own influences to control bias. Transcripts were not returned to the participants for member checking.

## Results

Seven focus groups before adapted cardiac rehabilitation included: 10 cardiac rehabilitation and stroke nurses (9 females), 14 cardiac rehabilitation and stroke physiotherapists (11 females), 6 consultants/medical stroke staff (3 females), 3 stroke occupational therapists (all female), 1 stroke physiotherapy assistants (male) and 1 stroke speech therapist (female).

Five focus groups after adapted cardiac rehabilitation included: 8 cardiac rehabilitation and stroke nurse specialists (7 females), 7 cardiac rehabilitation and stroke physiotherapists (all female), 2 stroke occupational therapists (both female), 2 stroke generic workers (both female), 2 cardiac rehabilitation exercise professionals (1 female) and 1 stroke speech therapist (female). The mean number of participants in each focus group was 5 (range 4–7). Overall, 57 health professionals participated in 12 focus groups (see Table 2 for composition of focus groups).

**Table 2.** Composition of focus groups.

Focus group	Moderators – number and names	Length in minutes	Number and type of participants	Place of Focus group
1 – CR staff before ACR	3 – NCB, SD, AH	43	6 = 3N, 3P	Glenfield hospital meeting room
2 – S staff before ACR	3 – NCB, AH, SD	71	5 = 1OT, 3P, 1GW	Leicester Royal hospital meeting room
3 – S staff before ACR	2 – NCB, SD	67	4 = 1D, 3P	Leicester Royal hospital meeting room
4 – CR staff before ACR	2 – NCB, AH	56	7 = 1P, 6N	Glenfield hospital meeting room
5 – S staff before ACR	2 – NCB, SD	54	5 = 2P, 1OT, 1D, 1N	Leicester General hospital meeting room
6 – S staff before ACR	2 – NCB, AH	62	4 = 4D	Leicester Royal hospital meeting room
7 – S staff before ACR	2 – NCB, SD	67	4 = 2PT, 1OT, 1 SALT	General hospital meeting room
8 – S staff after ACR	2 – NCB, AH	62	4 = 3P, 1N	Leicester General hospital meeting room
9 – CR staff after ACR	2 – NCB, SD	67	6 = 1P, 3N, 1EP	Glenfield hospital meeting room
10 – S staff after ACR	2 – NCB, SD	56	4 = 1N, 1OT, 1PT, 1GW	Leicester General hospital meeting room
11 – S staff after ACR	2 – NCB, AH	68	5 = 1OT, 1 GW, 1P, 1 N, 1 SALT	Leicester General hospital meeting room
12 – CR staff after ACR	2 – NCB, AH	53	4 = 2N, 1P, 1EP	Glenfield hospital meeting room

Key: CR – Cardiac rehabilitation; ACR – Adapted Cardiac Rehabilitation; S – Stroke; NCB – Nicola Clague-Baker; AH – Annegret Hagenberg; SD – Sophie Drewry; D – Doctor; P – physiotherapist; N – nurse; OT – Occupational therapist; SALT – speech and language therapist; GW – generic worker; EP – exercise professional.

There were no dropouts and all staff approached took part in the study. The average length of the focus groups was 60 minutes with a range of 43 to 71 minutes. Data saturation was achieved after the final focus group although it needs to be acknowledged that some of the stroke team was under-represented such as stroke nurses and speech therapists. The range of experience of cardiac rehabilitation in the Cardiac team was from 1 year to over 30 years and the range of experience of stroke in the Stroke team was from 1 year to over 20 years.

The subcategories identified by main theme in these focus groups were the following:

1. *Adapted cardiac rehabilitation for stroke survivors*: Positive impact of adapted cardiac rehabilitation, negative effect of adapted cardiac rehabilitation, referral considerations of adapted cardiac rehabilitation, barriers to adapted cardiac rehabilitation and cardiac rehabilitation adaptations;
2. *Confidence*: Cardiac rehabilitation teams and stroke survivors, stroke teams toward cardiac rehabilitation teams;
3. *Knowledge*: Cardiac rehabilitation teams and stroke survivors, stroke teams and cardiac rehabilitation, exercise and healthy lifestyles; and
4. *Healthy lifestyles and behaviour change*.

Themes 1 and 4 were identified in advance but themes 2 and 3 emerged from the data.

### Adapted cardiac rehabilitation for stroke survivors

#### Positive impact

After the adapted cardiac rehabilitation, all the cardiac rehabilitation staff felt that adapted cardiac rehabilitation had a positive influence on the stroke survivors, and stroke survivors had a positive impact on the cardiac patients. ‘*they (stroke participants) get a lot out of it*’ (cardiac rehabilitation focus group 5 after adapted cardiac rehabilitation), ‘*great improvement in confidence*’ (cardiac rehabilitation focus group 5 after adapted cardiac rehabilitation), ‘*positive effect on other people in the room*’ (cardiac rehabilitation focus group 2 after adapted cardiac rehabilitation). In addition, both teams identified that most patients were motivated early after stroke.

#### Negative impact

Prior to adapted cardiac rehabilitation, stroke therapists were concerned about the negative effect that adapted cardiac rehabilitation could have on high tone leading to more asymmetry and poor functional ability, ‘*because you can get associated reactions…so I think it does take careful management*’ (stroke focus group 2 before adapted cardiac rehabilitation), and ‘*from my aspect it's the tone*’ (stroke focus group 2 before adapted cardiac rehabilitation). However, the therapists who saw the patients after adapted cardiac rehabilitation found that their concerns were lessened.

The stroke teams and cardiac rehabilitation staff did identify that sometimes the stroke survivors felt self-conscious, particularly the more disabled and younger patients. ‘*Self-awareness as well, some patients are really quite conscious of how they might be moving differently*’ (stroke focus group 7 before adapted cardiac rehabilitation), ‘*there are issues with body image*’ (stroke focus group 6 before adapted cardiac rehabilitation), ‘*their anxiety and their self-consciousness*’ (cardiac rehabilitation focus group 5 after adapted cardiac rehabilitation).

#### Referral considerations

The stroke teams were instrumental in identifying and referring stroke survivors for adapted cardiac rehabilitation. They felt that the patients were particularly keen to get involved if the therapists were positive about adapted cardiac rehabilitation ‘*I think maybe presenting it as a positive thing did help*’ (stroke focus group 4 after adapted cardiac rehabilitation), and that it provided more therapy after the Early Supported Discharge teams finished ‘*it does leave a hole when we pull out and yes it was great to be able to offer them something else*’ (stroke focus group 3 after adapted cardiac rehabilitation).

#### Barriers

Both teams were aware of the psychological and environmental barriers to stroke survivors attending adapted cardiac rehabilitation and doing exercise. However, the stroke teams were aware of more physical and cognitive barriers, ‘*pre-existing cardiac problems, uncontrolled diabetes, blood pressure*’ (stroke focus group 1 after adapted cardiac rehabilitation), ‘*their communication…and also their cognitive skills*’ (stroke focus group 4 after adapted cardiac rehabilitation).

#### Adaptations

Both teams were very clear that in order for stroke survivors to attend adapted cardiac rehabilitation the main adaptation was having a specialist stroke physiotherapist in the class, ‘*you do need someone who has more experience dealing with strokes there for the patients’ benefit*’ (stroke focus group 2 before adapted cardiac rehabilitation) and ‘*I think there is an education need because it's 20 odd years since I properly looked after stroke survivors and that was back in my training*’ (cardiac rehabilitation focus group 1 before adapted cardiac rehabilitation). In addition, the teams felt that stroke teams would need to provide specialist education for the stroke population so they could answer any specific questions (Tables 3 and 4).

**Table 3.** Additional quotes related to adapted cardiac rehabilitation and exercise for stroke survivors.

Subcategory	Additional quotes
Positive impact of cardiac rehabilitation and exercise	‘*great improvement in confidence*’ (CRFG5A), ‘*they fitted in really well*’ (CRFG5A).
	‘*stroke patients put a lot more effort into the classes than a lot of cardiac patients do*’ (CRFG5A).
	Social: ‘*It’s about joining a group and seeing other people and talking to them. So there are a lot of things going on apart from just the exercise*’ (CRFG1B)
	Psychological: ‘*exercise makes you feel good*’ (SFG3B), ‘*It’s good for your mood*’ (SFG5B)
	Physical: ‘*reduces falls doesn’t it*’ (SFG6B), ‘*Blood pressure control I would have thought*’ (SFG5B), ‘*reduce their risk factors hopefully*’ (CRFG4B), ‘*Gain their independence*’ (CRFG4B), ‘*prevent a stroke happening again*’ (SFG2B).
Negative effect	Physical – ‘*we would need to know that the blood pressure was well controlled because exercise is going to raise their blood pressure*’ (CRFG1B), ‘*they have had a vascular event so there is some vascular risk*’ (SFG2B), ‘*I think one thing would be symmetry…..increasing tone*’ (CRFG1B), ‘*because you can get associated reactions…so I think it does take careful management*’ (SFG2)
	Psychological – ‘*quite scary to join a group of people that are established*’ (CRFG1B), ‘*might find it quite demoralising if everyone else is…*’ (SFG5B), ‘*I think some people are reluctant to exercise after an event because they think it’s going to happen again*’ (CRFG1B). ‘*self-conscious…and feel should I be here*’ (CRFG5A), particularly the younger participants who ‘*wouldn’t normally mix with these older people*’ (CRFG5A).
Barriers	Physical – ‘*falls risk*’ (SFG5B), ‘*Blood pressure, how controlled their diabetes is*’ (SFG3B), ‘*Physical ability*’ (SFG3B), ‘*inattentive to one side*’ (SFG2B), ‘*cognitive ability to follow instructions*’ (SFG2B)
	Psychological – ‘*motivation, depression those kind of things*’ (SFG7B), ‘*self-awareness as well, some patients are really quite conscious of how they might be moving differently*’ (SFG7B), ‘*There are issues with body image*’ (SFG6B), ‘*their anxiety and their self-consciousness*’ (CRFG5A), ‘*some people don’t want to be seen out in the community if they look different*’ (SFG3A).
	Environmental – ‘*transport issues, motivational issues*’ (CRFG2A), ‘*funding as well as transport*’ (SFG3A)

Key: CRFG – Cardiac rehab focus group; SFG – Stroke focus group; B – Before adapted cardiac rehabilitation; A – After adapted cardiac rehabilitation.

**Table 4.** Additional quotes related to adaptations of cardiac rehabilitation.

Subcategory	Additional quotes
Training of staff	‘*I would agree, having done 20 years in cardiology …we don’t have the background [in stroke]*’ (CRFG5A), ‘*[you need to be]… capable of doing a talk on different strokes*’. (CRFG5A), ‘… *…feel uncomfortable if it wasn’t closely monitored*’ (SFG1A) and ‘*There’s a whole lot more to look at with a stroke patient than with a cardiovascular patient*’ (SFG1A).
Specialist staff	‘*… difficult class to manage … without the support of the stroke physios*’ (CRFG2A), ‘*… feedback to get the best movement pattern*’ (SFG1A), ‘*… keep an eye on are they sticking to what they should be doing*’ (SFG1A),
Class	Content and equipment: ‘*we are getting a bike now, it’s not got a thing in the middle so they don’t have to get over it*’, ‘*whether they can actually physically get on a treadmill and walk on a treadmill …and if they are going to be doing weights can they manage on their weak side*’ (CRFG1B)
Education of patients	‘*education around stroke and medication*’ (CRFG4B), ‘*it would be worth talking about education about strokes and how long it could take people to improve, the dips and the plateaus and the challenges they could face*’ (SFG7B)

Key: CRFG – Cardiac Rehabilitation Focus group; SFG – Stroke focus group; B – Before adapted cardiac rehabilitation; A – After adapted cardiac rehabilitation.

### Confidence

Confidence was a major theme for cardiac rehabilitation and stroke staff. Stroke teams lacked confidence in the cardiac rehabilitation teams; some commented that ‘*I would feel uncomfortable if (the tone) wasn’t closely monitored*’ (stroke focus group 4 after adapted cardiac rehabilitation), ‘*I think it's important to have a stroke specialist in there because we see people every day that have had strokes but they’re not the same as each other*’ (stroke focus group 3 after adapted cardiac rehabilitation), and ‘*I was reassured by the fact there was a stroke physio there*’ (stroke focus group 1 after adapted cardiac rehabilitation).

The cardiac rehabilitation staff initially lacked confidence with stroke survivors saying: ‘*it's just a massive learning curve*’ (cardiac rehabilitation focus group 4 after adapted cardiac rehabilitation), and ‘*we didn’t know what we didn’t know*’ (cardiac rehabilitation focus group 4 after adapted cardiac rehabilitation). However, by the end of the research study, some of the cardiac rehabilitation staff felt ‘*I am more confident now I think than at the start*’ (cardiac rehabilitation focus group 5 after adapted cardiac rehabilitation) and ‘*much much better than I thought it was going to be. An enjoyable experience*’ (cardiac rehabilitation focus group 5 after adapted cardiac rehabilitation) although they still commented that ‘*I think you definitely need a stroke nurse or a stroke physio*’ (cardiac rehabilitation focus group 4 after adapted cardiac rehabilitation)

### Knowledge

Cardiac teams wished they had more background knowledge on stroke management, e.g., splints, ‘*I didn’t have a clue did I and I felt very vulnerable there* (related to splint)’ (cardiac rehabilitation focus group 2 after adapted cardiac rehabilitation), and quality of movement, ‘*because of our lack of knowledge we wouldn’t have even picked that up*’ (cardiac rehabilitation focus group 2 after adapted cardiac rehabilitation). Stroke teams felt unqualified to give advice on healthy lifestyle, ‘*I don’t think we have enough experience or confidence in giving that advice* (healthy lifestyle)’ (stroke focus group 1 after adapted cardiac rehabilitation), and advised stroke survivors to read Stroke Association leaflets, but overall wished to have more training in adapted cardiac rehabilitation, ‘*I don’t exactly know what they do when they go there. I know they do exercise*’ (stroke focus group 4 after adapted cardiac rehabilitation).

It also appeared that some stroke team members had a lack of knowledge of the importance of cardiac rehabilitation exercise saying, ‘*That's why I like the active lifestyle exercise referrals …sitting doing some tai chi or a chair-based exercise*’ (stroke focus group 3 after adapted cardiac rehabilitation). When asked how they recommend stroke survivors build up their fitness, one response was ‘*class-based exercise…there's lots of seated exercise groups*’ (stroke focus group 1 after adapted cardiac rehabilitation), indicating a lack of knowledge of the intensity needed to increase cardiorespiratory fitness. Stroke teams that treated stroke survivors in the acute phase of recovery felt that exercise and getting fit was not a priority at this stage, ‘*priorities up to that point was not about getting fit, it's been about getting out of bed*’ (stroke focus group 1 after adapted cardiac rehabilitation), and they felt that discussing exercise at this stage was ‘*almost a bit insensitive*’ (stroke focus group 1 after adapted cardiac rehabilitation).

Stroke teams also appeared to be more reluctant to encourage the use of the term ‘exercise’, preferring the term ‘activity’:

‘I don’t ever use the term exercise when I am talking to a patient…because if you say exercise patients assume that's going for a run or going to the gym’ (stroke focus group 2 before adapted cardiac rehabilitation).

Whereas cardiac rehabilitation teams highlighted the importance of exercise rather than activity: ‘*people think some of their activities are exercise when it's not*’ (cardiac rehabilitation focus group 4 before adapted cardiac rehabilitation).

### Healthy lifestyles and behaviour change

Cardiac rehabilitation teams were very clear that education about healthy lifestyle and behaviour change was a definite part of their role, ‘*we all have a role*’ (cardiac rehabilitation focus group 2 after adapted cardiac rehabilitation), ‘*we’re the foundations*’ (cardiac rehabilitation focus group 2 after adapted cardiac rehabilitation) and ‘*we’re sowing the seeds*’ (cardiac rehabilitation focus group 2 after adapted cardiac rehabilitation). They felt that the stroke survivors were positive towards healthy lifestyle messages, ‘*I think they’re interested in healthy eating*’ (cardiac rehabilitation focus group 2 after adapted cardiac rehabilitation), and ‘*have a good attitude to changing lifestyles to get better*’ (cardiac rehabilitation focus group 2 after adapted cardiac rehabilitation).

Stroke teams in the later stage of rehabilitation also appeared to acknowledge their role in healthy lifestyle advice: ‘*I certainly do use the leaflets and there is information about healthy diet*’ (stroke focus group 3 after adapted cardiac rehabilitation). However, they felt that ‘*because you are in their home you can’t really, it's difficult to turn around and preach to them*’ (stroke focus group 3 after adapted cardiac rehabilitation). Acute stroke teams stated that ‘*we don’t properly sit down and talk to anybody about lifestyle*’ (stroke focus group 1 after adapted cardiac rehabilitation), and they felt they do not have the time or opportunity.

They also appeared more negative towards patients taking up behaviour change: ‘*I don’t think it's high on their agenda*’ (stroke focus group 1 after adapted cardiac rehabilitation), and ‘*I don’t think I see many that make that change*’ (stroke focus group 1 after adapted cardiac rehabilitation). In the stroke teams, only certain professions seemed to provide healthy lifestyle messages, which was reflected in a comment by one of the speech and language therapists,

‘I think it hasn’t been that high on my list of things that I’m thinking about and it should be being part of a stroke team…maybe I should be bringing that in as part of my role’ (stroke focus group 4 after adapted cardiac rehabilitation).

Both teams identified a number of barriers that impacted on behaviour change, including financial pressures, stress, anxiety, and faulty beliefs about exercise:

‘*they are unemployed and they have money problems…they can’t give up smoking because of that…stress levels and anxiety*’ (cardiac rehabilitation focus group 4 before adapted cardiac rehabilitation). However, it appeared that the cardiac rehabilitation team were more familiar with the process of health behaviour change, identifying key factors to support this, such as: confidence-building, education and goal-setting: ‘*We do try and influence them to have goals based upon the information that we have…personalise it…based on information about their cholesterol, weight or exercise*’ (cardiac rehabilitation focus group 4 before adapted cardiac rehabilitation).

## Discussion

The main themes identified in these focus groups were: delivery of adapted cardiac rehabilitation for stroke survivors; Confidence; Knowledge; and Healthy lifestyles and behaviour change. Cardiac rehabilitation teams were very positive towards stroke survivors taking part in adapted cardiac rehabilitation. They identified a number of benefits for both stroke and cardiac patients, which was not recognised by the stroke teams who did not see the stroke survivors after discharge. The stroke teams appeared to lack confidence in the cardiac rehabilitation teams’ ability to provide appropriate advice and physical and technical support for the stroke stroke survivors. This needs to be recognised as the positive attitude of referrers to cardiac rehabilitation programmes has a beneficial impact on the attendance levels to cardiac rehabilitation and pulmonary rehabilitation programmes.^19,20^

It was identified that one negative effect of the adapted cardiac rehabilitation programme was the psychological impact on more severely disabled stroke survivors who were self-conscious and embarrassed about their disabilities. This needs to be taken into account when deciding on stroke survivors to include into existing cardiac rehabilitation programmes. Whilst National Institutes of Health Stroke Score^17^ scores of 0 to 5 are considered mild strokes, this could still include individuals with significant physical impairment who may need close supervision, so it will be important to discuss with potential candidates for adapted cardiac rehabilitation.

Another issue considered by the stroke teams was the potential impact on high tone. It is identified that up to 43% of stroke survivors develop post-stroke spasticity,^21^ and traditionally it has been thought that this may be worsened by increased activity and effort. However, the teams did not report an increase in tone over the six weeks of adapted cardiac rehabilitation, and so stroke teams were less concerned about referring patients into the programmes.

Both teams identified environmental barriers, such as lack of transport, as previously reported.^22^ This needs to be considered when providing adapted cardiac rehabilitation, as all stroke survivors are unable to drive for at least one month following stroke, as identified mandated by the Driver and Vehicle Licensing Agency.^23^ In addition, cardiac rehabilitation teams did not identify as many physical and cognitive barriers for stroke survivors attending adapted cardiac rehabilitation, compared to stroke teams. However, having taken part in the adapted programmes, some of the cardiac rehabilitation teams had an increased awareness of the specific needs of stroke survivors, such as fatigue and tonal needs.

Both teams expressed (prior to and after adapted cardiac rehabilitation) the need for a specialist stroke physiotherapist to attend the adapted cardiac rehabilitation programmes to advise on appropriate adaptations to exercise and to provide stroke advice and education, in particular for physical and cognitive difficulties. This would have an impact on the level of disability or stroke severity that can be included in existing cardiac rehabilitation programmes.

Cardiac rehabilitation teams identified a lack of knowledge of and confidence with stroke survivors and felt they would need more education and support if stroke survivors were to be included in the adapted cardiac rehabilitation programmes. This was reinforced by the stroke teams being more aware of additional aspects such as fatigue, falls and shoulder damage. As up to 85% of stroke survivors suffer fatigue,^24^ up to 73% fall post-stroke^25^ and up to 22% suffer shoulder pain post-stroke,^26^ it is imperative that everyone treating stroke survivors is aware of these difficulties and knows how to manage them. This lack of awareness is an important consideration if cardiac rehabilitation programmes were to support stroke survivors in cardiac rehabilitation classes, especially as the British Association for Cardiovascular Prevention and Rehabilitation national audit^27^ identified that only 63% of programmes had physiotherapists on their staff. It also highlights the need to consider the different levels of ability before placing stroke survivors in cardiac rehabilitation programmes.

Whilst stroke teams recognised the importance of specific cardiorespiratory training and healthy lifestyle for the stroke population, and the need for the whole team to emphasise this healthy message, they lacked the knowledge of the cardiac rehabilitation staff, as identified in previous studies.^12,13,15^ In addition, the healthy lifestyle messages were not seen as a priority in acute stroke, in contrast to cardiac rehabilitation teams; again, confirming previous studies.^28^ However, cardiac rehabilitation services provide these messages in the early stages of cardiac recovery, highlighting the importance of lifestyle risk management and health behaviour change as two of the seven core components of cardiac rehabilitation defined by the British Association for Cardiovascular Prevention and Rehabilitation.^27^ Behaviour change models, such as the transtheoretical model,^29^ have identified that there are five stages of behaviour change: pre-contemplation, contemplation, pre-preparation, action and maintenance. People immediately post-stroke may be more likely to contemplate behaviour change, so the sooner the messages are provided to them and their families the more likely behaviour change will occur. However, Mauk et al.^30^ suggests the Mauk model post-stroke identifying that stroke survivors may be more likely to take on advice in the later stages of recovery. This would indicate the importance of stroke teams, particularly early supported discharge teams, receiving more education on how to provide healthy lifestyle messages and how to facilitate behaviour change.

It is important to recognise some limitations of the study. First, not all members of the stroke and cardiac rehabilitation teams were represented, despite the researchers endeavouring to recruit a wide range of staff. Secondly, there was potential bias using healthcare professionals as facilitators and data analysers. However, the variety of beliefs and experience within the team ensured rigour.

Future research needs to further explore staff attitudes towards exercise, healthy lifestyle and behaviour change provision after stroke particularly at different stages of recovery and with different levels of stroke severity.

In conclusion, cardiac rehabilitation and stroke staff attitudes to adapted cardiac rehabilitation for stroke survivors showed a range of benefits, negatives, barriers and adaptations needed. One of the main adaptations was for the cardiac rehabilitation service to be supported by a specialist stroke physiotherapist if it were to include people with mild stroke. Confidence and knowledge of the cardiac rehabilitation team to support mildly disabled stroke survivors, and confidence and knowledge of the stroke teams to provide healthy lifestyle advice and behaviour change techniques, needs to be addressed with training if this approach was to be applied to clinical practice.

**Clinical messages**

1. Cardiac rehabilitation teams recognise the benefits of adapted cardiac rehabilitation for stroke survivors with mild disability but acknowledge they need more specialist support and training.
2. Stroke teams identify a lack of understanding of and confidence in adapted cardiac rehabilitation for stroke survivors which could be addressed with training.
